# Detection of Aortic Dissection and Intramural Hematoma in Non-Contrast Chest Computed Tomography Using a You Only Look Once-Based Deep Learning Model

**DOI:** 10.3390/jcm13226868

**Published:** 2024-11-14

**Authors:** Yu-Seop Kim, Jae Guk Kim, Hyun Young Choi, Dain Lee, Jin-Woo Kong, Gu Hyun Kang, Yong Soo Jang, Wonhee Kim, Yoonje Lee, Jihoon Kim, Dong Geum Shin, Jae Keun Park, Gayoung Lee, Bitnarae Kim

**Affiliations:** 1 Department of Convergence Software, Hallym University, Chuncheon 24252, Republic of Korea; yskim01@hallym.ac.kr (Y.-S.K.); dainee96@gmail.com (D.L.); kongjw0110@gmail.com (J.-W.K.); 2 Department of Emergency Medicine, Kangnam Sacred Heart Hospital, Hallym University College of Medicine, Seoul 07441, Republic of Korea; gallion01@hallym.ac.kr (J.G.K.); emkang@hallym.or.kr (G.H.K.); amicoys@hallym.or.kr (Y.S.J.); wonsee02@hallym.or.kr (W.K.); yoonje@hallym.or.kr (Y.L.); 3 Hallym Biomedical Informatics Convergence Research Center, Kangnam Sacred Heart Hospital, Hallym University College of Medicine, Seoul 07441, Republic of Korea; 43583@hallym.ac.kr (G.L.); bnaraekim12@hallym.ac.kr (B.K.); 4 Department of Thoracic and Cardiovascular Surgery, Kangnam Sacred Heart Hospital, Hallym University College of Medicine, Seoul 07441, Republic of Korea; jkim@hallym.or.kr; 5 Division of Cardiology, Department of Internal Medicine, Kangnam Sacred Heart Hospital, Hallym University College of Medicine, Seoul 07441, Republic of Korea; blau07@hallym.or.kr; 6 Department of Internal Medicine, Kangnam Sacred Heart Hospital, Hallym University College of Medicine, Seoul 07441, Republic of Korea; jaekpark@hallym.or.kr; 7 Department of Health Policy and Management, Ewha Womans University Graduate School of Clinical Biohealth, Seoul 03760, Republic of Korea

**Keywords:** aortic dissection, tomography, contrast media, deep learning, machine learning, artificial intelligence

## Abstract

**Background/Objectives**: Aortic dissection (AD) and aortic intramural hematoma (IMH) are fatal diseases with similar clinical characteristics. Immediate computed tomography (CT) with a contrast medium is required to confirm the presence of AD or IMH. This retrospective study aimed to use CT images to differentiate AD and IMH from normal aorta (NA) using a deep learning algorithm. **Methods**: A 6-year retrospective study of non-contrast chest CT images was conducted at a university hospital in Seoul, Republic of Korea, from January 2016 to July 2021. The position of the aorta was analyzed in each CT image and categorized as NA, AD, or IMH. The images were divided into training, validation, and test sets in an 8:1:1 ratio. A deep learning model that can differentiate between AD and IMH from NA using non-contrast CT images alone, called YOLO (You Only Look Once) v4, was developed. The YOLOv4 model was used to analyze 8881 non-contrast CT images from 121 patients. **Results**: The YOLOv4 model can distinguish AD, IMH, and NA from each other simultaneously with a probability of over 92% using non-contrast CT images. **Conclusions**: This model can help distinguish AD and IMH from NA when applying a contrast agent is challenging.

## 1. Introduction

Aortic dissection (AD) and aortic intramural hematoma (IMH) are life-threatening conditions that require prompt diagnosis and treatment. Their clinical characteristics and therapeutic strategies are similar, and contrast-enhanced computed tomography (CT) is crucial for confirmation. AD and IMH have incidences of 2.6–3.5 cases per 100,000 person-years. Therefore, patients with these conditions are not commonly seen in emergency departments but do occasionally seek treatment [1,2,3]. Endovascular treatment for aortic disorders, particularly acute aortic syndrome, has become increasingly popular in recent years, and survival rates have increased [4]. However, the mortality rate of untreated type A AD and IMH increases by 1–2% per hour after symptom onset, and the acute-phase mortality rate is high [5]. Therefore, emergency physicians must consider AD and IMH in the initial differential diagnosis for patients who visit the emergency department with severe chest pain.

Contrast-enhanced CT is considered to be the best diagnostic tool because of its affordability, speed, and high specificity and sensitivity. According to data from the International Registry of Acute Aortic Dissection, 63% of respondents selected CT as their first diagnostic modality [5]. If there is a possibility of AD or IMH, an urgent contrast-enhanced CT is required. However, if there is a history of anaphylactic reaction to contrast agents or a high risk of renal failure, deciding whether to use contrast media can be challenging [6,7].

Non-contrast CT is less time-consuming than contrast-enhanced CT media and can be performed in most emergency departments without waiting for serum creatinine levels to be confirmed, which often takes over an hour. However, this method may not be clinically relevant unless the diagnostic accuracy of non-contrast chest CT for AD and IMH is high.

Differentiating AD and IMH from the normal aorta (NA) using only non-contrast CT images in critical situations could enable faster treatment, increasing patient survival rates. Attempts have been made to detect AD using CT without contrast enhancement in order to overcome these issues in artificial intelligence (AI) and deep learning [8], but simultaneous detection of AD and IMH has not yet been researched. Since IMH and AD share common symptoms and require rapid diagnosis and treatment, the ability to simultaneously differentiate AD and IMH in non-contrast CT would be clinically significant for emergency physicians. Deep learning using non-contrast CT images would allow the development of an algorithm that accurately identifies AD and IMH from NA without a contrast agent.

## 2. Methods

### 2.1. Study Design and Setting

This retrospective study used CT images to differentiate AD and IMH from NA using a deep learning algorithm. From January 2016 to July 2021, CT images were obtained from a university hospital in Seoul in the Republic of Korea. The Institutional Review Board (IRB) of our hospital approved this study (IRB no. 2021-08-013); since this was a retrospective study, informed consent was not required. Identifying information was removed from each image before the onset of the study. All methods and procedures were performed in accordance with the Declaration of Helsinki.

### 2.2. Selection of Participants

CT images were categorized into three classes: AD, IMH, and NA. The AD and IMH groups were selected from patients according to the International Classification of Diseases 10th Revision ICD-10 I71.01, dissection of the thoracic aorta and I71.01, dissection of the aorta, unspecified, from the hospital’s electronic medical records. Four experts in emergency medicine (EM), one in cardiothoracic surgery (CS), and one in cardiovascular medicine examined patients’ medical records and chest angiography CT images. Two radiology specialists included patients identified as having AD or IMH. In this study, we obtained and included CT images from the aortic root to the iliac artery bifurcation. The NA group comprised 50 randomly selected cases using the same modality (chest angiography CT), where no abnormalities in the aorta or major arteries were observed. Patients aged <18 years, those with AD or IMH caused by trauma, and those with significant motion artifacts on CT images were excluded. Cases in which the position and morphology of the aorta differed from normal cases, such as those with prior aortic surgery, lobectomy, aortic aneurysm, or dextrocardia, were also excluded. CT images were acquired using Somatom Definition Flash 256 (Siemens, Munich, Germany) and Somatom Sensation 64 (Siemens, Munich, Germany). The selected images were extracted from the patients’ images in jpg format using the INFINITT M6 Picture Archiving and Communication System (PACS) (INFINITT Healthcare, Seoul, Republic of Korea), which is linked to the electronic medical record. The images underwent resizing, normalization, and augmentation. Windowing, which is the process of modifying the CT scan grayscale via the CT numbers (Hounsfield Units), was performed on the images to help highlight key anatomical features for easier recognition. As a result, the metadata Window Width and Window Center values of the images were adjusted to enhance the contrast appearance of the features.

Furthermore, the image size was resized to 512 × 512 pixels to estimate anchor boxes, and the data were normalized between 0 and 1 to maximize data integrity. The mosaic method, which combines four images into one in certain ratios, was used for augmentation. This allows the model to identify objects in smaller scales, thus, improving the generalization of the object detection task.

### 2.3. Model Development for CT Image-Based AD and an IMH Detection Algorithm

Using the included 8881 images (AD, n = 2965; IMH, n = 1688; and NA, n = 4228) from 121 patients, we first located the aorta on CT images. The You Only Look Once version 4 (YOLOv4) framework was used to determine whether the differentiated aorta contained AD or IMH. NA, AD, and IMH are the class types of the aorta detection data, which were learned using the MakeSense tool “https://www.makesense.ai (accessed on 15 September 2024)” by entering the location coordinates and labels of the bounding box (Figure S1). Images assigned to the test set were not included in the training set to avoid overfitting and generalizability.

After all independent images were collected, the images were divided into training, validation, and test sets in an 8:1:1 ratio. Training, validation, and testing were performed to determine whether AD or IMH was present on images.

The machine learning process had three stages. (1) CT images used in the first step of the study were tagged to the aorta using the YOLO program. (2) The image and labeled data values were used for the training process to locate the aorta on the CT images. (3) Training was performed to confirm the presence or absence of lesions and the type of lesions in the aorta discovered in the previous step.

#### 2.3.1. Aorta Labeling on Chest CT Images and Data Processing

For masking, a researcher painted the entire aortic area on chest CT images in agreement with another for aorta segmentation. Non-contrast and postcontrast CT images from the AD and IMH groups were extracted separately and then coupled at the same level. Because AD and IMH are difficult to identify in non-contrast images, each image was classified as AD, IMH, or NA by assessing the presence of AD or IMH lesions on each non-contrast image based on the paired post-contrast images. All this work was performed by EM specialists. In case of disagreement between the two EM reviewers, a third reviewer (EM or CS) was consulted; differences were resolved through discussion until a consensus was reached.

#### 2.3.2. Object Detection Model

**Figure 1:**
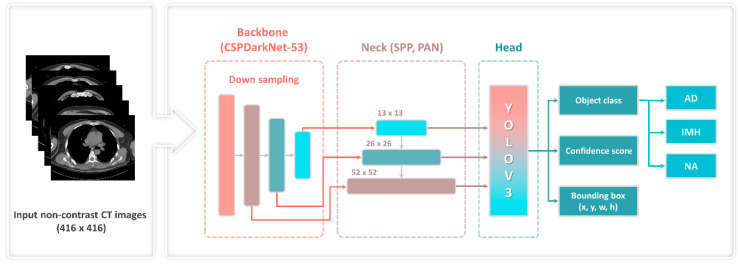
YOLOv4 framework with CSP-Darknet53. AD—aortic dissection; CT—computed tomography; CSP—cross-stage partial; IMH—aortic intramural hematoma; NA—normal aorta; PAN—path aggregation network; SPP—spatial pyramid pooling; YOLOv3—You Only Look Once version 3.

The 1-stage detector YOLOv4, which comprises a backbone, neck, and head, and simultaneously predicts classes and bounding boxes, was used in the object detection model (Figure 1). The input image was converted into a feature map using the backbone. The feature map extracted from the backbone was localized to the head. That is, it contributes to locating the class and bounding boxes of the image. The neck, which connects the backbone to the head, reconstructs and refines the feature map. Additionally, a bag of freebies (BoFs) and a bag of specials (BoSs) were applied to improve the model’s performance. The BoFs method uses data augmentation techniques, regulation, and loss and can improve performance without increasing costs. BoS includes a skip connection, a feature pyramid network, and an activation function, which increases the cost while improving performance.

#### 2.3.3. Main Characteristics of the YOLOv4 Framework

Figure 1 illustrates the overall structure of the YOLOv4 object detection model. The backbone generated feature maps using a down-sampling technique and a pretrained cross-stage partial (CSP)-Darknet53 neural network with an input image of 416 × 416 pixels. CSP decreased (by ~20%) the computation required by a convolutional neural network. Subsequently, the neck, comprising the spatial pyramid pooling and path aggregation network, connected feature maps of various scales (13, 26, 52) and refined the data. The existing YOLOv3 was used to predict the location of the bounding box, object class, and confidence score of the final head.

BoF used Cutmix, mosaic, and label smoothing in the model as data augmentation techniques, whereas drop-block regulation and label smoothing were used as regulation techniques. Moreover, the Complete Intersection over Union (CIOU) loss, which measures the similarity between predicted and ground-truth bounding boxes, is applied via the loss function. CIOU loss aims to provide a more accurate localization loss by addressing aspect ratio and box overlap issues [9].

In this study, 8881 CT images were used to implement the YOLOv4 object detection model as training (80%), validation (10%) and testing (10%) datasets. We classified the images not at the patient level, but at the image level for AD, IMH, and NA. Non-contrast chest CT images obtained from a single patient comprise approximately 70–100 images, depending on the patient’s physique. Even if a patient is diagnosed with AD or IMH, only some of these images contain the lesion, while the rest show an NA. If images were labeled as AD, IMH, or NA at the patient level instead of the image level during training, it could lead to incorrect learning. Images without AD or IMH could be misclassified due to the diagnosis of AD or IMH. Therefore, to prevent such issues, we labeled whether AD or IMH was present and conducted training and testing at the image level. Each image was assigned three class names (AD, IMH, and NA) and the aortic bounding box locations. The gradient optimization function used was the momentum stochastic gradient descent; the batch size was 64 during network training. The number of epochs was set to 2000 for each class and 6000 in total.

#### 2.3.4. Main Metrics for the Detection Algorithm of AD and IMH

The most popular metrics for object detection models were used: mean average precision (*mAP*), Intersection Over Union (*IoU*), and precision–recall (P-R) curve. In object detection frameworks, true negatives (TNs) are not used [10]. TN represents a correctly undetected nonexistent object; in object detection, there are an infinite number of bounding boxes that should not be detected within the image [11]. Therefore, any TN-based metric, such as receiver operating characteristic curve, accuracy, or specificity, was avoided.

In object detection tasks, *mAP* represents the average precision (*AP*) across all categories, where ${AP}_{i}$ represents the *AP* value of the *i*th class, *N* represents the total number of classes, and *AP* and *mAP* have values within the range [0, 1].

(1) $$mAP=\frac{1}{N}\sum_{i=1}^{N}{AP}_{i}$$

*AP* is a comprehensive index that considers precision and recall. Since precision and recall have values between 0 and 1, *mAP* also has a value between 0 and 1.

In classification tasks with localization and object detection, the *IoU* (Equation (2)) is frequently used to determine the reliability of the bounding box location, where prediction ($B_{P}$) and ground truth ($B_{GT}$) are the predicted and ground truth bounding boxes, respectively [12]. *IoU* equals zero for non-overlapping bounding boxes and one for perfect overlap.

(2) $$IoU=\frac{area\left(B_{P}\cap B_{GT}\right)}{area\left(B_{P}\cup B_{GT}\right)}$$

The *IoU* threshold was set to 0.5. An *IoU* > 0.5 was considered accurate, and precision and recall values were determined. As shown in Equations (3) and (4), the *AP* for a specific class is calculated by interpolating all points. Consequently, a P-R curve was developed to determine the *AP* of all recall values.

(3) $$AP=\sum_{n}\left(R_{n+1}-R_{n}\right)P_{interp}\left(R_{n+1}\right)$$

(4) $$P_{interp}\left(R_{n+1}\right)=\max\;P\left(\tilde{R}\;:\tilde{R}\geq \;R_{n+1}\right)$$

The P-R curve displays the precision in accordance with the modification of the recall value produced by modifying the confidence score. This is a technique for assessing the object detection model’s performance, which is typically calculated as the area under the P-R curve.

Below are the mathematical definitions of precision, recall, and F_1_-score.

(5) $$Precision=\frac{True\;Positive}{True\;Positive+False\;Positive}$$

(6) $$Recall=\frac{True\;Positive}{True\;Positive+False\;Negative}$$

(7) $$F_{1}\text{-}\mathrm{score}=\frac{2\cdot Precision\cdot Recall}{Precision+Recall}$$

### 2.4. Analysis

Data were compiled using a standard spreadsheet application (Excel 2016; Microsoft, Redmond, WA, USA) and analyzed using SPSS 20 (SPSS Inc., Chicago, IL, USA). The Kolmogorov–Smirnov test was performed to evaluate the distribution normality of all datasets. Descriptive statistics are presented as frequencies and percentages for categorical data and either a median and interquartile range (nonnormal distribution) or a mean and standard deviation (normal distribution) for continuous data. We used Student’s *t* test or the Mann–Whitney U test to compare the characteristics of segmented and non-segmented data. *p*-values of <0.05 were considered statistically significant. All statistical calculations were performed using Python and R software (version 3.4.1; “https://www.r-project.org (accessed on 15 September 2024)”.

## 3. Results

### 3.1. Characteristics of the Study Subjects

**Figure 2:**
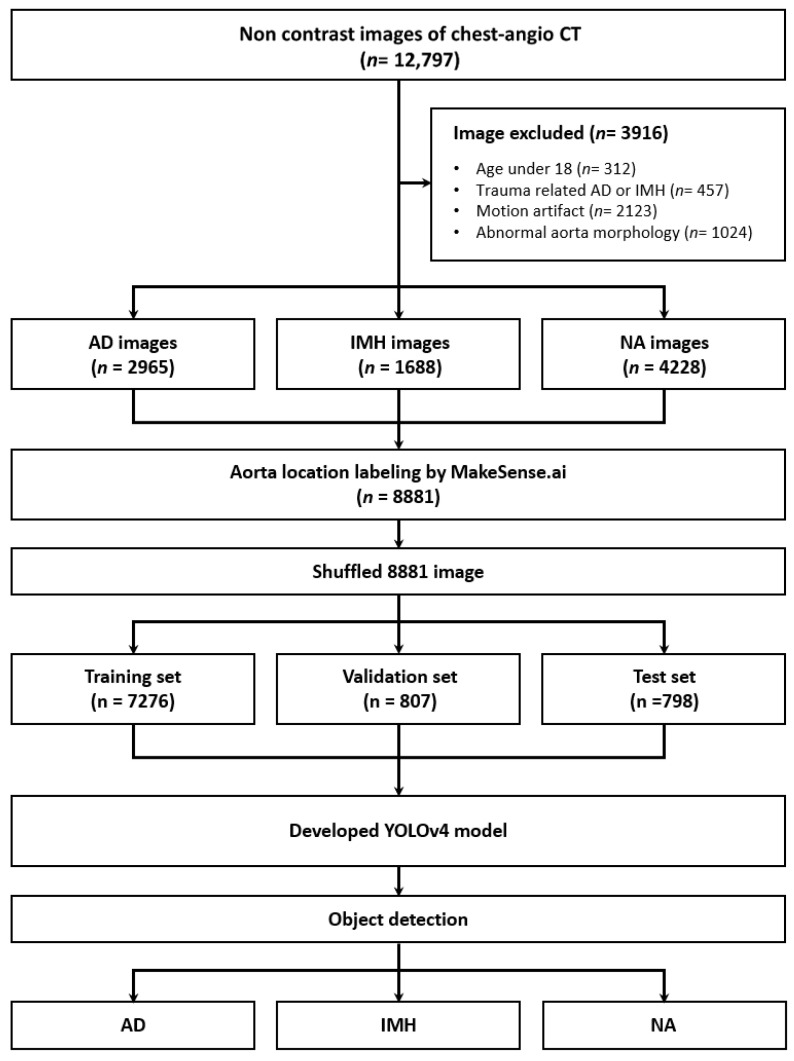
Flow diagram of the study. CT—computed tomography; AD—aortic dissection; IMH—aortic intramural hematoma; NA—normal aorta; YOLOv4—You Only Look Once version 4.

In total, 12,797 images were obtained from 121 patients, 72 of whom were male (59.5%), with a median age of 63.0 (50.0–73.0). Based on the exclusion criteria, 3916 images were excluded. Finally, 8881 images, including 2965 of AD and 1688 of IMH, were included (Figure 2). Among the variables, except for hypertension, no significant differences were observed between the groups (all *p* > 0.05). Although there were substantial differences in BMI among groups, the retrospective study’s limitations resulted in numerous missing values (55/121, 45.5%). (Table 1).

**Figure 3:**
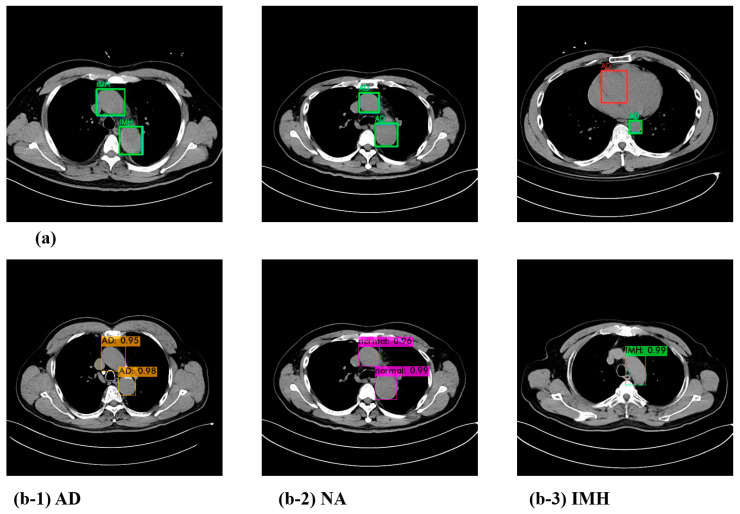
Visualization of the prediction result (**a**) and test results with the confidence score of the YOLOv4 model (**b**). (**b-1**) AD; (**b-2**) NA; (**b-3**) IMH. AD—aortic dissection; IMH—aortic intramural hematoma; NA—normal aorta.

The YOLOv4 model outputs the predicted object class, confidence score, and bounding box coordinates when making the final prediction at the head. The confidence score is calculated as the “probability of an object class × *IoU*”. When conducting an experiment to detect the presence or absence of a dissection in the aorta, an example test output of the model is shown in Figure 3b, where the bounding box and confidence score for the detected location and class can be confirmed.

Furthermore, we compared the predicted results of the model with the actual ground truth class and the bounding box. Figure 3a depicts cases wherein the model made accurate predictions as well as those in which it did not. The blue box represents the ground truth, the green box represents the predicted result, and the red box represents the box that the detector misidentified. Since the CT images for each case used in the test comprised sequential data, it was possible to detect and correct such sparse errors.

### 3.2. Model Performance of the Algorithm of AD and IMH Detection

**Figure 4:**
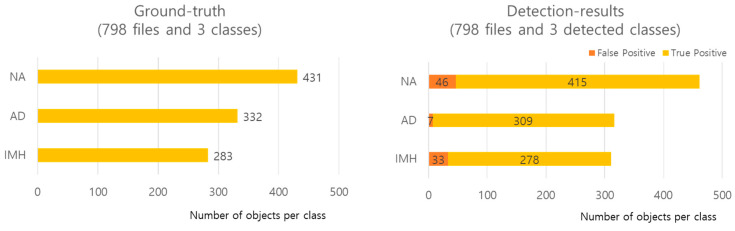
Distribution of each class and detailed detection results. AD—aortic dissection; IMH—aortic intramural hematoma; NA—normal aorta.

In the experiment aimed at detecting NA, AD, and IMH on CT images, of the 8881 images extracted from 121 patients, 7276 were used for the training set, 807 were used for the validation set, and 798 were used for the test set. The three class distributions and detailed detection results (false positive [FP] and true positive [TP]) of the model using the test data are shown in Figure 4. TP indicates correct detection when the *IoU* is greater than the threshold value; FP indicates positive detection when the *IoU* is smaller than the threshold value. Cases without detection were excluded, resulting in a difference between ground truth and predicted results.

**Figure 5:**
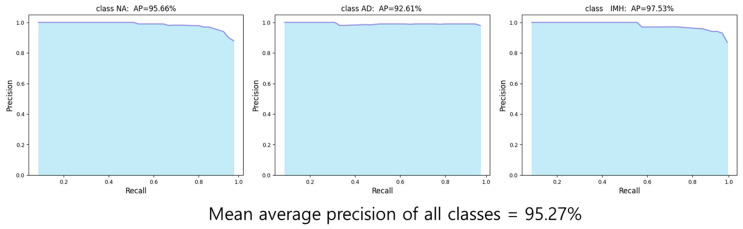
Average precision graph (P-R curve) for each class. AD—aortic dissection; *AP*—average precision; IMH—aortic intramural hematoma; NA—normal aorta.

The final test results of the YOLOv4 model for AD and IMH detection, based on the evaluation metrics for object detection, showed an *AP* of 95.66%, 92.61%, and 97.53% for NA, AD, and IMH, respectively (Figure 5). The final *mAP* for all classes was 95.27%.

**Figure 6:**
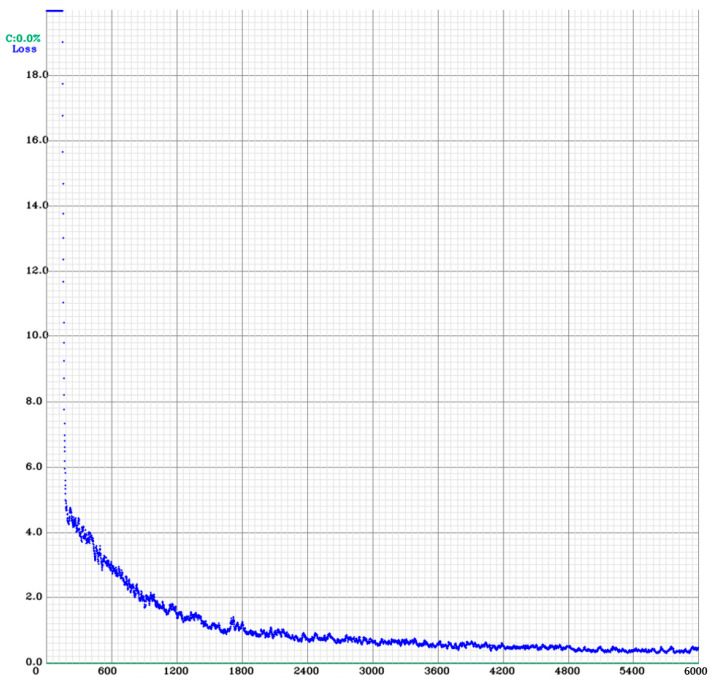
Results of the YOLOv4 training. CIOU—Complete Intersection over Union; CIOU loss curve graph; YOLOv4—You Only Look Once version 4.

Figure 6 shows a descending trend in the CIOU loss curve, indicating that the model is learning and improving its ability to predict the bounding boxes accurately.

## 4. Discussion

We developed a deep learning-based algorithm that can accurately detect AD and IMH using non-contrast-enhanced CT images. The proposed YOLOv4 model demonstrated an *AP* of 95.66% for NA, 92.61% for AD, and 97.53% for IMH.

The diagnosis of AD and IMH is generally based on imaging tests [13]. By necessity, in addition to CT with contrast agents, MR and TEE are also commonly used as imaging examination methods. Compared to CT with contrast agents, MR offers the advantage of having less side effects, lowering radiation exposure, and being the best alternative examination for patients with CT contraindications [14]. TEE is significant in intraprocedural decision-making, as it helps to decrease complications of thoracic endovascular aortic repair (TEVAR) and improve survival rates. However, in emergency settings, contrast-enhanced CT scans are still commonly used [15].

AD and IMH treatment involves aggressive blood pressure control, and surgical intervention may be necessary to repair or replace affected aortic segments. Owing to rapid progression of AD and IMH, early and accurate diagnosis is crucial [16,17]. Recent studies have shown that an AI-based algorithm for detecting AD is more effective than human interpretation of chest CT scans [8]. Nevertheless, this AI study was limited in its ability to detect IMH on CT scans, since it did not include IMH in the training of AI-based algorithms. Although AD and IMH have different underlying pathophysiology, they share many similar symptoms and require prompt medical attention.

Clinicians, especially emergency physicians, may have a reduced diagnostic ability in scenarios where patients present with atypical symptoms or when patient influx to the emergency room is high, leading to time constraints. However, the YOLOv4 model could provide consistent and stable performance even in asymptomatic patients without limiting reading time, potentially improving the accuracy and efficiency of AD and IMH diagnoses [18,19,20]. The YOLOv4 model used in this study may also aid clinical decision-making by alerting nonspecialist clinicians to cases with suspected AD or IMH. It can also indicate specific slice levels suspicious of AD or IMH, enabling clinicians to review the identified images in more detail.

Contrast-enhanced CT for the diagnosis of AD and IMH can lead to complications such as anaphylactic reaction to the contrast agent, contrast-induced nephropathy (CIN), and skin necrosis or vasculitis resulting from contrast agent leakage into the extravascular area [6,7]. Since contrast agents are excreted through the kidneys, emergency physicians confirm adequate renal function in patients before conducting contrast-enhanced CT. However, in urgent cases where prompt use of contrast is imperative to differentiate AD or IMH from NA, physicians must weigh the benefits and risks of using contrast before verifying renal function. Using the YOLOv4 model, emergency physicians could reduce delays in CT scan time and unnecessary complications, such as anaphylactic reactions or CIN caused by contrast exposure from enhanced CT.

The YOLOv4 model is a deep learning framework that is widely used for real-time object detection in images and videos. Its advantages include speed, accuracy, and versatility, making it useful for various object detection applications such as autonomous vehicles, surveillance systems, and robotics [21]. Overall, for this research, YOLOv4 is a powerful object detection model that can achieve state-of-the-art performance while maintaining real-time performance [21,22,23].

In AD or IMH, accurate imaging interpretation is crucial in diagnostic and exclusion processes, as observed symptoms can also be present in other conditions, such as acute coronary syndrome [24,25]. Contrast-enhanced CT not only confirms AD or IMH diagnosis but also provides information on the type of dissection and the extent of involvement in the vessels that supply blood to the brain, heart, kidneys, and other organs, helping determine the appropriate treatment approach [24,25]. Therefore, although the AI algorithm cannot replace contrast-enhanced CT in AD and IMH diagnosis, the proposed YOLOv4 model minimizes the unnecessary use of contrast media and helps diagnose patients with suspected AD or IMH using only non-contrast images, even before verifying the serum creatinine level results, which can take more than an hour. Moreover, the overall *AP* calculated for the images was >95%. When serial non-contrast CT images from a single patient are sequentially applied to the YOLOv4 model, and if AD or IMH is detected in multiple consecutive images, the accuracy of diagnoses by emergency physicians will improve.

This study has some limitations. First, chest CT images and patient data were obtained from a single center, and our proposed model has not been validated in other hospital settings. Therefore, further investigation using non-contrast chest CT images from various hospitals is needed. Second, we did not assess how different aortic conditions influenced our model’s results. Third, we did not compare the performance of the model and physicians with respect to key factors, such as clinical outcomes and the equipment required to use the model as a screening tool. Fourth, this study did not perform an analysis concerning BMI, which could influence image quality [26]. Fifth, we excluded images that had significant motion artifacts from our study. Because of their instability, critically ill patients with AD and IMH may have more motion artifacts than ordinary patients. As a result, we believe that additional study is required to determine the accuracy of AD and IMH diagnosis using images of patients with motion artifacts, as well as to develop algorithms that can substantially increase the accuracy. Finally, we did not include penetrating aortic ulcers (PAUs). Acute aortic syndrome includes PAUs, along with AD and IMH. However, we excluded PAUs from our study because of insufficient imaging data.

## 5. Conclusions

Deep learning can improve the detection accuracy for AD and IMH in non-contrast CT images. Our results can help rapidly screen unstable patients with suspected AD or IMH in the emergency department and those with a history of anaphylaxis or CIN risk.

## Figures and Tables

**Table 1:** Basic patient characteristics.

Variables	Total	NA	AD	IMH	*p*
	(N = 121)	(N = 47)	(N = 49)	(N = 25)	
Age, years	63.0 [50.0–73.0]	60.0 [49.5–77.0]	63.0 [56.0–69.0]	69.0 [42.0–77.0]	0.566
Sex					0.164
Male	72 (59.5%)	23 (48.9%)	32 (65.3%)	17 (68.0%)	
Female	49 (40.5%)	24 (51.1%)	17 (34.7%)	8 (32.0%)	
Stanford type					-
Type A	16 (13.2%)	-	10 (20.4%)	6 (24.0%)	
Type B	58 (47.9%)	-	39 (79.6%)	19 (76.0%)	
HTN					<0.001
No	44 (36.4%)	28 (59.6%)	12 (24.5%)	4 (16.0%)	
Yes	77 (63.6%)	19 (40.4%)	37 (75.5%)	21 (84.0%)	
DM					0.072
No	93 (76.9%)	32 (68.1%)	38 (77.6%)	23 (92.0%)	
Yes	28 (23.1%)	15 (31.9%)	11 (22.4%)	2 (8.0%)	
CKD					0.164
No	118 (97.5%)	44 (93.6%)	49 (100.0%)	25 (100.0%)	
Yes	3 (2.5%)	3 (6.4%)	0 (0.0%)	0 (0.0%)	
Operation					0.050
No	114 (94.2%)	46 (97.9%)	43 (87.8%)	25 (100.0%)	
Yes	5 (4.1%)	0 (0.0%)	5 (10.2%)	0 (0.0%)	
Unknown	2 (1.7%)	1 (2.1%)	1 (2.0%)	0 (0.0%)	
BMI (kg/m^2^)					0.001
Underweight (BMI < 18.5)	2 (1.7%)	1 (2.1%)	1 (2.0%)	0 (0.0%)	
Normal weight (BMI ≥ 18.5 to 24.9)	17 (14.0%)	9 (19.1%)	4 (8.2%)	4 (16.0%)	
Overweight (BMI ≥ 25 to 29.9)	47 (38.8%)	9 (19.1%)	30 (61.2%)	8 (32.0%)	
Obesity (BMI ≥ 30)	0 (0.0%)	0 (0.0%)	0 (0.0%)	0 (0.0%)	
Unknown	55 (45.5%)	28 (59.6%)	14 (28.6%)	13 (52.0%)	

AD—aortic dissection; CKD—chronic kidney disease; DM—diabetes mellitus; HTN—hypertension; IMH—aortic intramural hematoma; NA—normal aorta; BMI—body mass index.

## Data Availability

The datasets used and/or analyzed during the current study are available from the corresponding author on reasonable request.

## Supplementary Materials

The following supporting information can be downloaded at https://www.mdpi.com/article/10.3390/jcm13226868/s1, Figure S1: Depiction of a masked aortic CT image using MakeSense. CT, computed tomography.
