# Supplementary material for: Detection of Aortic Dissection and Intramural Hematoma in Non-Contrast Chest Computed Tomography Using a You Only Look Once-Based Deep Learning Model
Source: J Clin Med. 2024 Nov 14;13(22):6868. doi: 10.3390/jcm13226868 (PMC11594775; doi:10.3390/jcm13226868)
Supplement: Supplementary file 1 [file jcm-13-06868-s001.zip › jcm-3260131-supplementary.pdf]

Article

Detection of Aortic Dissection and Intramural Hematoma in Non-Contrast Chest CT Using YOLO-Based Deep Learning Model

Yu-Seop Kim [1,†], Jae Guk Kim [2,3,†], Hyun Young Choi [2,3,*], Da-in Lee [1], Jin-Woo Kong [1], Gu Hyun Kang [2,3], Yong Soo Jang [2,3], Wonhee Kim [2,3], Yoonje Lee [2,3], Jihoon Kim [4], Dong Geum Shin [5], Jae Keun Park [6], Gayoung Lee [3,7] and Bit-narae Kim [3]

Supplementary Materials:

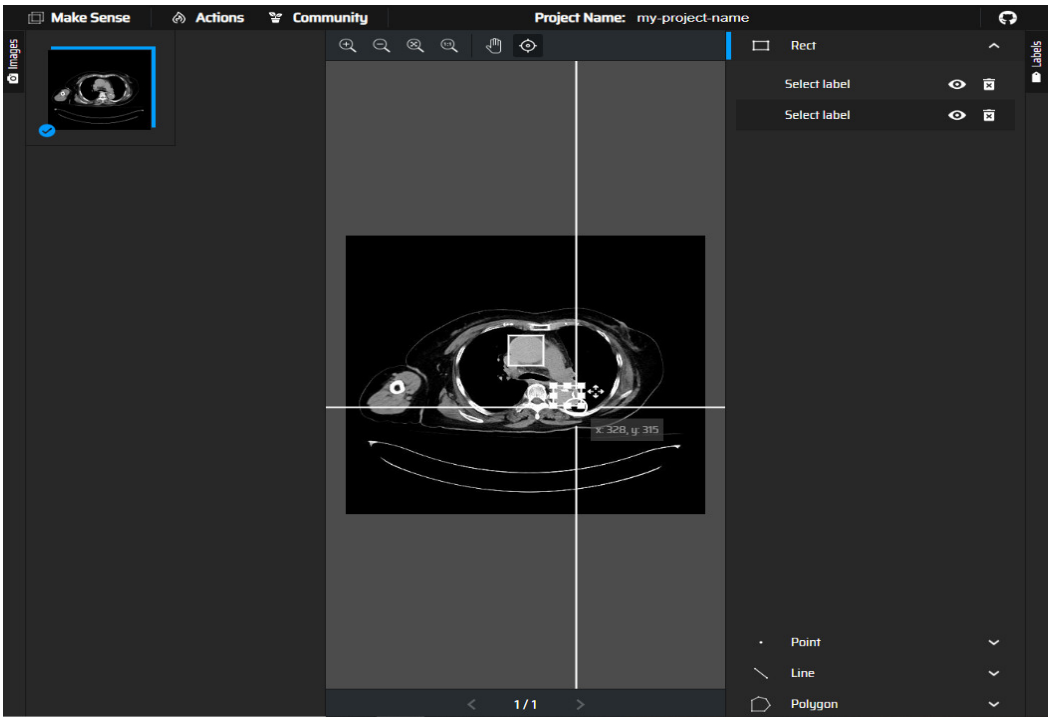

Figure S1. Depiction of a masked aortic CT image using MakeSense.

CT, computed tomography.
